# Specified Species in Gingival Crevicular Fluid Predict Bacterial Diversity

**DOI:** 10.1371/journal.pone.0013589

**Published:** 2010-10-25

**Authors:** Sirkka Asikainen, Başak Doğan, Zeynep Turgut, Bruce J. Paster, Aysen Bodur, Jan Oscarsson

**Affiliations:** 1 Section of Oral Microbiology, Institute of Odontology, Umeå University, Umeå, Sweden; 2 Department of Periodontology, Faculty of Dentistry, Marmara University, Istanbul, Turkey; 3 Department of Periodontology, Gazi University, Ankara, Turkey; 4 Department of Molecular Genetics, The Forsyth Institute, Boston, Massachusetts, United States of America; 5 Department of Oral Medicine, Infection and Immunity, Harvard School of Dental Medicine, Boston, Massachusetts, United States of America; Charité Universitätsmedizin Berlin, Germany

## Abstract

**Background:**

Analysis of gingival crevicular fluid (GCF) samples may give information of unattached (planktonic) subgingival bacteria. Our study represents the first one targeting the identity of bacteria in GCF.

**Methodology/Principal Findings:**

We determined bacterial species diversity in GCF samples of a group of periodontitis patients and delineated contributing bacterial and host-associated factors. Subgingival paper point (PP) samples from the same sites were taken for comparison. After DNA extraction, 16S rRNA genes were PCR amplified and DNA-DNA hybridization was performed using a microarray for over 300 bacterial species or groups. Altogether 133 species from 41 genera and 8 phyla were detected with 9 to 62 and 18 to 64 species in GCF and PP samples, respectively, per patient. Projection to latent structures by means of partial least squares (PLS) was applied to the multivariate data analysis. PLS regression analysis showed that species of genera including *Campylobacter, Selenomonas, Porphyromonas, Catonella*, *Tannerella*, *Dialister, Peptostreptococcus*, *Streptococcus* and *Eubacterium* had significant positive correlations and the number of teeth with low-grade attachment loss a significant negative correlation to species diversity in GCF samples. OPLS/O2PLS discriminant analysis revealed significant positive correlations to GCF sample group membership for species of genera *Campylobacter, Leptotrichia, Prevotella, Dialister, Tannerella, Haemophilus*, *Fusobacterium, Eubacterium,* and *Actinomyces*.

**Conclusions/Significance:**

Among a variety of detected species those traditionally classified as Gram-negative anaerobes growing in mature subgingival biofilms were the main predictors for species diversity in GCF samples as well as responsible for distinguishing GCF samples from PP samples. GCF bacteria may provide new prospects for studying dynamic properties of subgingival biofilms.

## Introduction

Periodontitis remains widespread in populations aged 40 years or older [1] and represents one of the most common chronic bacterial infections in man. During its elongated progression, this asymptomatic infection causes local impairment, but it may also render the patients to increased risk for nonoral infections as well as to elevated proinflammatory host responses [2] linked to systemic diseases, such as cardiovascular diseases [3], [4]. The mechanisms how periodontitis induces systemic inflammation are still unclear, but may include systemic dissemination of live bacteria and components from lysed bacteria and, as recently suggested, free-soluble components from live planktonic and biofilm bacteria from periodontal pockets [5], [6].

Bacterial colonization and growth on supra- and subgingival tooth surfaces causes chronic inflammation in periodontal tissues. Increased flow of gingival crevicular fluid (GCF) [7], a serum transudate or inflammatory exudate, washes periodontal pockets and thereby provides host-derived substances that shape subgingival bacterial populations. Enriched with bacterial material from subgingival space GCF finally enters the oral cavity at the periodontal pocket orifice. In contrast to well-studied host-associated constituents of GCF [8], [9], no previous knowledge was found of its bacterial composition.

Recent advances in molecular methods give new possibilities to identify species in complex bacterial communities such as subgingival biofilms. Open-ended molecular biological methods have revealed the presence of previously unrecognized species and bacterial groups in subgingival samples [10], [11], [12], [13]. The collected 16S rDNA sequence data serve as a basis for developing rapid methods, such as DNA microarrays for bacterial detection and identification from clinical samples. A recently-launched DNA microarray provides simultaneous detection of hundreds of cultivable and not-yet-cultured bacterial species from oral samples [14], [15].

Passive and active detachment of bacterial cells from biofilms [16] occurs as part of their biofilm life cycle [17]. Detached (planktonic) bacteria downregulate genes required for biofilm formation and upregulate expression of virulence properties for an acute infection [18]. Thus, the quality and quantity of unattached bacterial populations in periodontal pockets and the phase of periodontal infection may depend on each other. Identifying unattached bacteria may also provide insight into the activity of subgingival biofilm *in vivo*. In the present study, we propose that bacterial species found in GCF reflect the bacterial populations detached from subgingival biofilms. As no previous knowledge was found of bacteria in GCF samples, our main objectives were to detect and identify bacterial species and to delineate contributors to species diversity in GCF samples. The results demonstrated complex bacterial populations. Certain species/phylotypes predicted species diversity in GCF samples and discriminated GCF samples from subgingival paper point (PP) samples.

## Results

Descriptive data on the demographic, systemic and dental variables of the periodontitis patients are presented in Table 1. From the total of 438 bacterial probe targets incorporated in the DNA microarray, 133 were included in the present data analyses. These 133 probe targets represent those that were detected in at least one patient in GCF and/or PP samples at levels higher than the HOMIM signal level score of 1 (Positive scores range from the lowest 1 to the highest 5). The mean weights of the GCF and PP samples per patient were 7.8±2.6 ug and 5.7±2.8 ug, respectively.

**Table 1:** Descriptive data of patients with periodontitis.

Variables		Mean ± SD
Age (years)		46.6±8.1
Gender (females; N, %)		6 (35.3)
Socioeconomic status (N, %)		
	Low	1 (5.9)
	Intermediate	11 (64.7)
	High	5 (29.4)
Smoking (N, %)		
	Never	7 (41.2)
	Stopped	3 (17.6)
	Current	7 (41.2)
Body Mass Index (kg/m^2^)		24.6±2.3
Leukocyte count (x10^9^/l)		7.3±1.3
Blood glucose (mg/dl)		85.5±8.2
Number of teeth		23.3±3.4
All teeth		
	Plaque Index	1.4±0.4
	Gingival Index	1.3±0.2
	Bleeding on probing (%)	39.2±17.9
	Probing depth (mm)	3.2±0.3
	Clinical attachment level (mm)	3.8±0.3
Sampled sites	Plaque Index	1.4±0.5
	Gingival Index	1.4±0.3
	Bleeding on probing (%)	84.7±23.6
	Probing depth (mm)	6.1±0.7
	Clinical attachment level (mm)	6.7±0.8

### Specified bacterial species but few host-associated variables contributed significantly to the species diversity in gingival crevicular fluid samples

**Figure 1:**
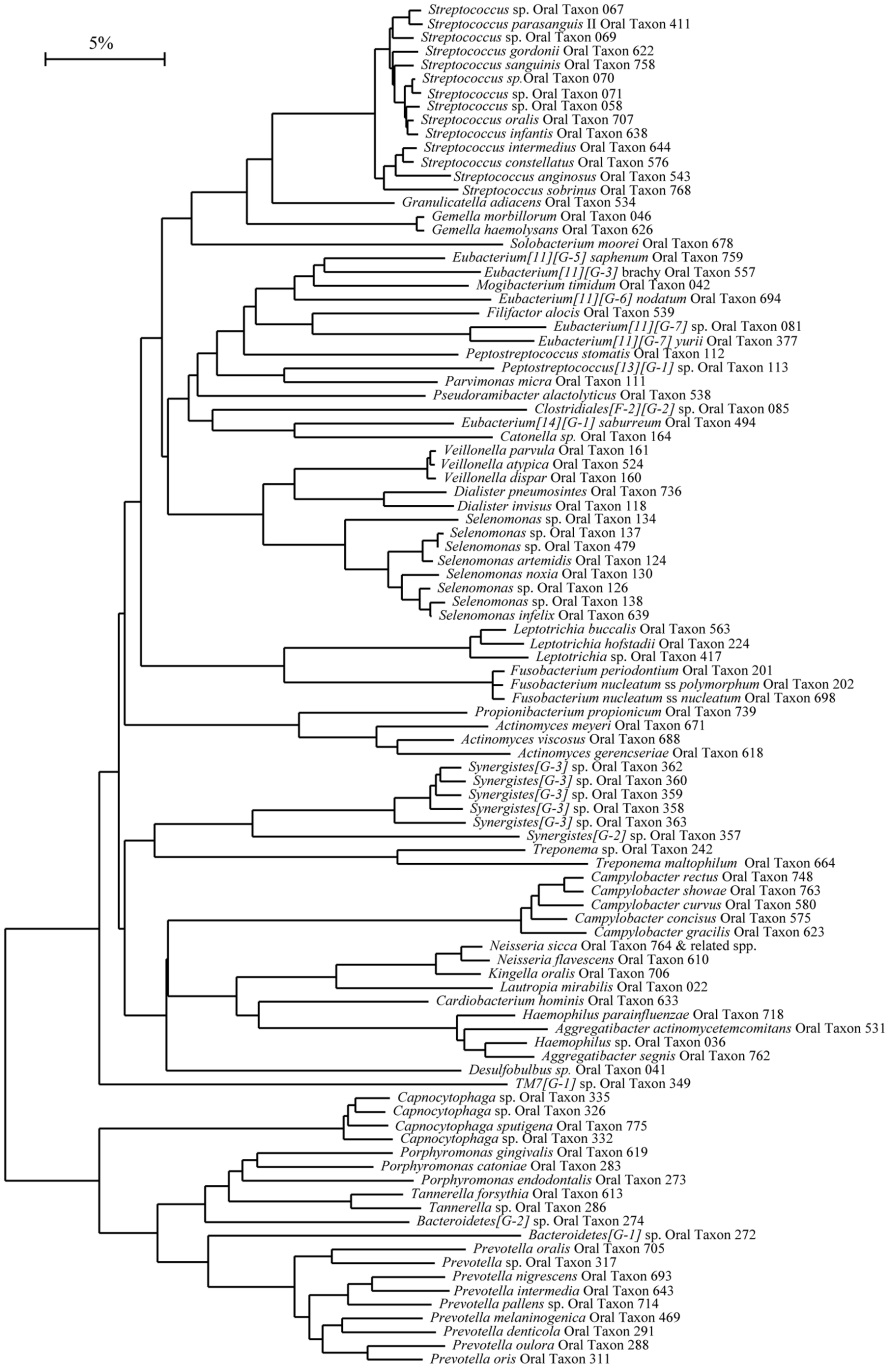
Phylogenetic tree of the bacterial species/phylotypes identified in DNA microarray analysis of GCF samples. The marker bar represents 5% difference in nucleotide sequences. GCF: gingival crevicular fluid.

**Figure 2:**
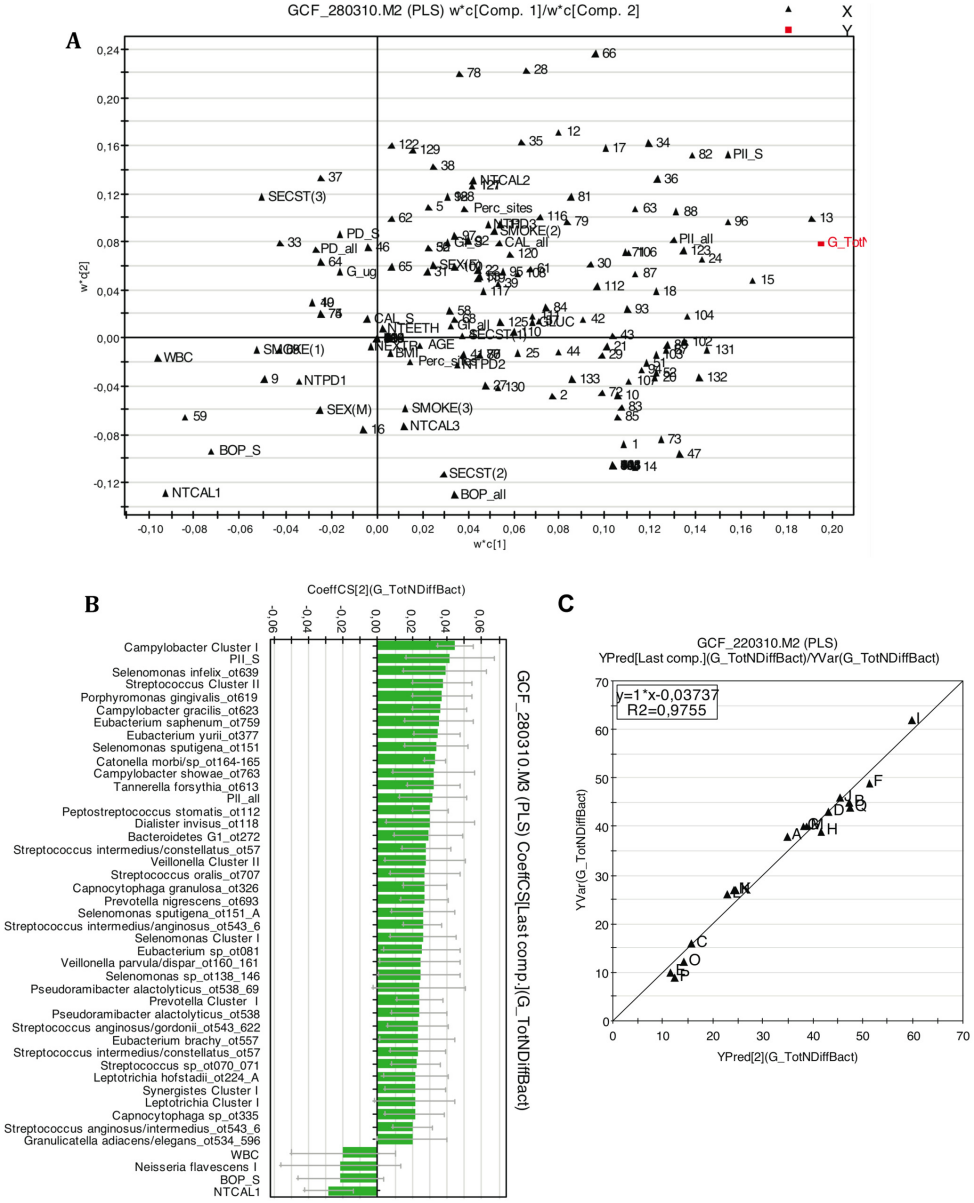
Interrelations between bacterial and host-associated variables and correlation to bacterial species/phylotype diversity in GCF samples. A multivariate PLS modeling was used for data analysis. Loading scatter plot (Panel A) displays the correlation structure of the variables (X variables: N = 166; Y variable: Number of different bacterial species/phylotypes in GCF samples). A number code was given for each bacterial taxon in their alphabetical order (Panel A) (the key is shown in online Supporting information Table S1). PLS regression coefficient plot (Panel B) identified X variables with statistically significant correlation to Y. The coefficients (>0.02 or <−0.02 are shown) are statistically significant when the error bars do not cross the 0 line. The model explained 98% and, according to cross validation, predicted 79% of the variation in Y. Observed values vs. predicted values R^2^ = 0.975 (Panel C). Capital letters are patient identifiers (Panel C). X variables were scaled and centered and Y variable scaled. The confidence intervals were derived from jack knifing. GCF: gingival crevicular fluid.

A phylogenetic analysis of the bacterial species identified in GCF samples revealed 8 bacterial phyla/candidate phyla, *Firmicutes, Bacteroidetes, Fusobacteria, Proteobacteria, Actinobacteria, Spirochaetes, Synergistetes* and TM7 (Figure 1). The number of different bacterial species (species diversity) in GCF samples ranged from 9 to 62 (mean 33.7, SD 15.3) per patient. In order to delineate which of the bacterial and host-associated study variables contributed to the species diversity per sample, we generated a multivariate PLS (Projection to latent structures by means of partial least squares) model using 166 X variables (133 bacterial species and 33 host-associated variables) and one Y variable (number of different species per sample) (Figure 2A, B, C). The loading scatter plot (Figure 2A) gives an overview of all variables; the position of each X variable shows its relationship to other X variables but also to the Y variable. The bacterial species are shown as numbers for which the key is given in online Supporting information, Table S1. The observed vs predicted values for the number of different species per sample demonstrated a good fit for the model (R^2^ = 0.975). According to cross validation the model predicted 79% of the variation in Y.

In the loading scatter plot (Figure 2A), the X variables closest to the Y variable suggests their positive correlation to each other and to species/phylotype diversity. They mainly included members of 3 phyla, *Proteobacteria*, *Firmicutes* and *Bacteroidetes*. Few host-related variables were found close to Y. Conversely, in the diagonal corner of the loading plot, suggesting a negative correlation to species/phylotype diversity, several host-associated variables were found in addition to members of 2 of the above phyla, i.e., *Proteobacteria* and *Bacteroidetes.*

A PLS regression analysis was additionally performed to evaluate the extent each X variable contributed to the bacterial species/phylotype diversity in GCF samples. After removing X variables with regression coefficients between 0.02 and −0.02 regarded as less important correlations, the remaining X variables with the highest positive and negative correlations are shown in Figure 2B. Significant positive correlations were found for species of genera belonging to 3 phyla, *Firmicutes* (*Selenomonas, Catonella, Dialister, Veillonella, Eubacterium, Peptostreptococcus, Pseudoramibacter* and *Streptococcus*), *Bacteroidetes* (*Porphyromonas, Prevotella* and *Tannerella*) and *Proteobacteria* (*Campylobacter*). Most of the streptococcal species were milleri (anginosus) group streptococci, *S. intermedius, S. constellatus* and *S. anginosus*. Only a single species, *Neisseria flavescens,* had a negative correlation (<−0.02) to species/phylotype diversity, although not significant at the 95% confidence interval level (Figure 2B).

Among the dental variables, Plaque Index (PlI) per patient at sampled sites and at all examined sites in the dentition was the only significant positive contributor to the species/phylotype diversity in GCF samples (Figure 2B). Periodontal variables, such as number of teeth with clinical attachment level (CAL) 4–6 mm (NTCAL2) or with probing depth (PD) >6 mm (NTPD3), mean CAL at all sites (CAL_all), proportion of sites with PD >6 mm, and Gingival Index (GI) at sampled sites (GI_S), as well as some other host-related variables e.g., glucose level and interrupted smoking habit, correlated positively, but not at 95% confidence interval level (data not shown). Conversely, the number of teeth with CAL <4 mm (NTCAL1) was the only significant negative contributor to species/phylotype diversity (Figure 2B). Mean frequency of bleeding on probing (BOP) at sampled sites (BOP_S) and leukocyte count had negative regression coefficients (<−0.02), but they did not reach statistical significance.

### Comparison between bacterial species in gingival crevicular fluid samples and subgingival paper point samples

**Figure 3:**
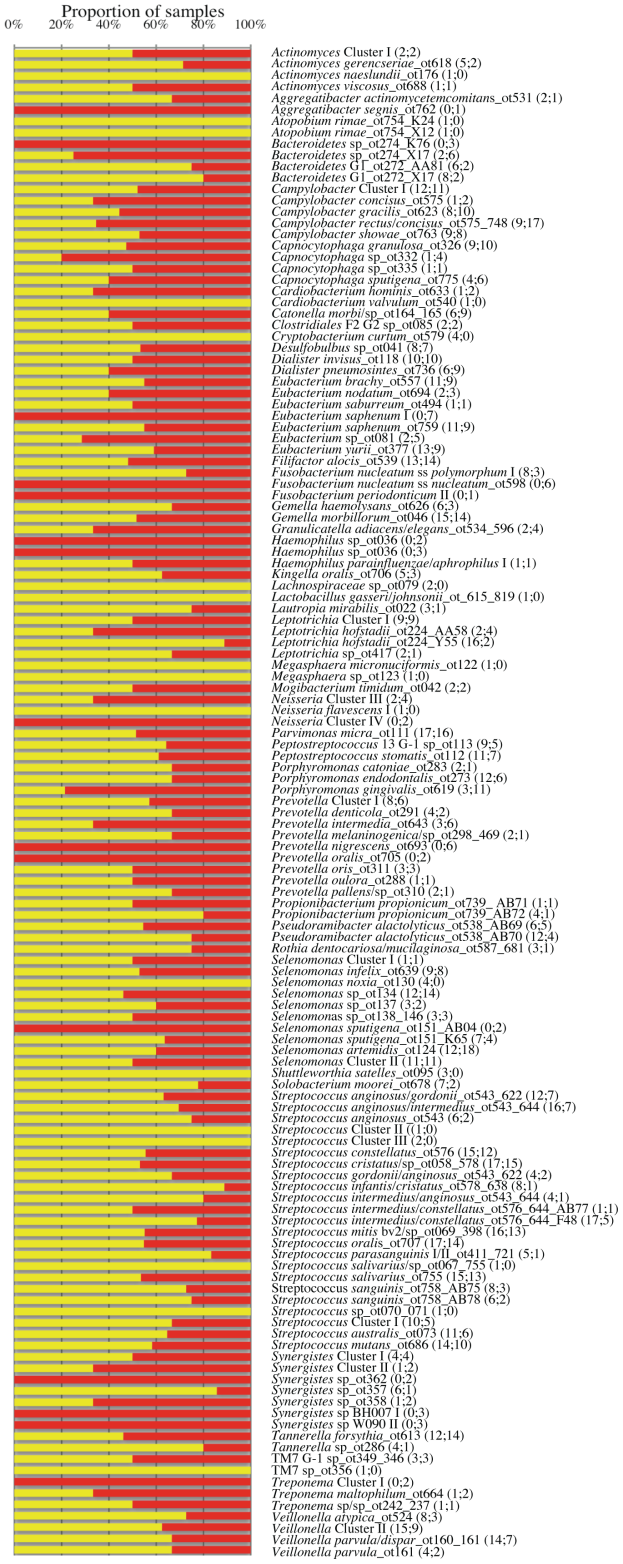
Distribution pattern for species/phylotypes in GCF samples and subgingival PP samples. The bars show proportion of GCF and PP samples of all samples testing positive for the depicted species/phylotypes. The numbers in parentheses after species definitions show the number of positive samples in both types of samples (PP;GCF). Yellow bars PP samples, red bars GCF samples. PP: paper point; GCF: gingival crevicular fluid.

We subsequently asked whether the composition of the bacterial populations identified in GCF samples differed from those identified in PP samples obtained from the same periodontal sites as the GCF samples. The overall species/phylotype diversity in PP samples, ranging from 18 to 64 bacterial species (mean 41.8, SD 12.0) per patient, resembled that in GCF samples (data above). Figure 3 demonstrates the distribution pattern of the 133 species or groups shown as detection frequency and percentage of all samples positive for the depicted species/phylotype in the GCF and PP samples, separately. Except for 15 (11%) species found only in GCF samples and 17 (13%) species found only in PP samples, the majority (N = 101, 76%) of the 133 species/phylotypes were identified in both sample types; 23 (23%) more often in GCF than PP samples and 58 (57%) more often in PP than GCF samples (Figure 3).

**Figure 4:**
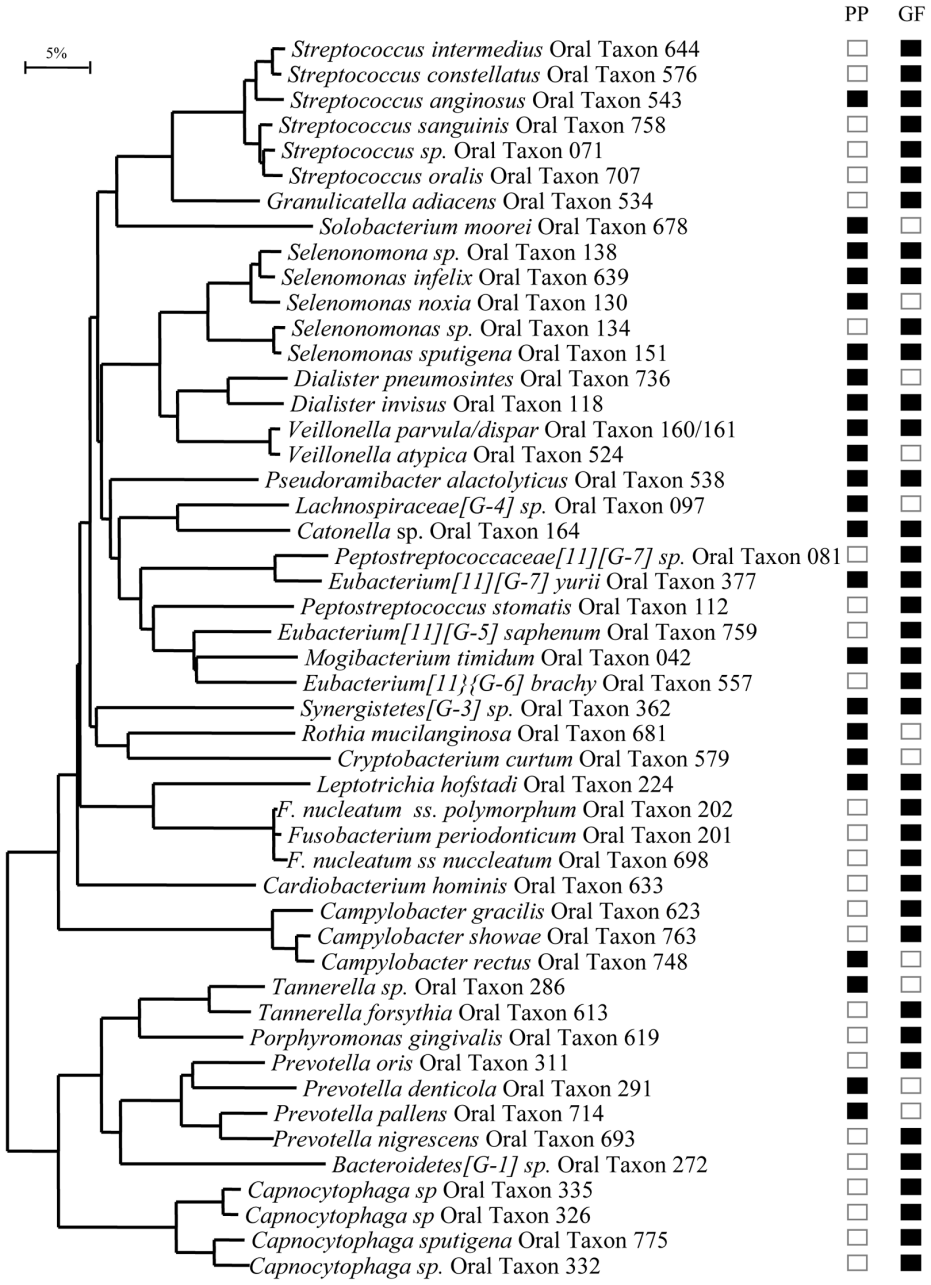
Phylogenetic analysis of bacterial species/phylotypes contributing to species/phylotype diversity in GCF samples and PP samples. The species/phylotypes with statistically significant PLS regression coefficients >0.02 or <−0.02 (data from Figure 2B for the GCF samples and from Figure 5 for the PP samples) were selected for the phylogenetic analysis. The marker bar represents 5% difference in nucleotide sequences. GF abbreviation for gingival crevicular fluid and PP for subgingival paper point samples. Black boxes indicate presence and white boxes absence of the depicted species/phylotype.

**Figure 5:**
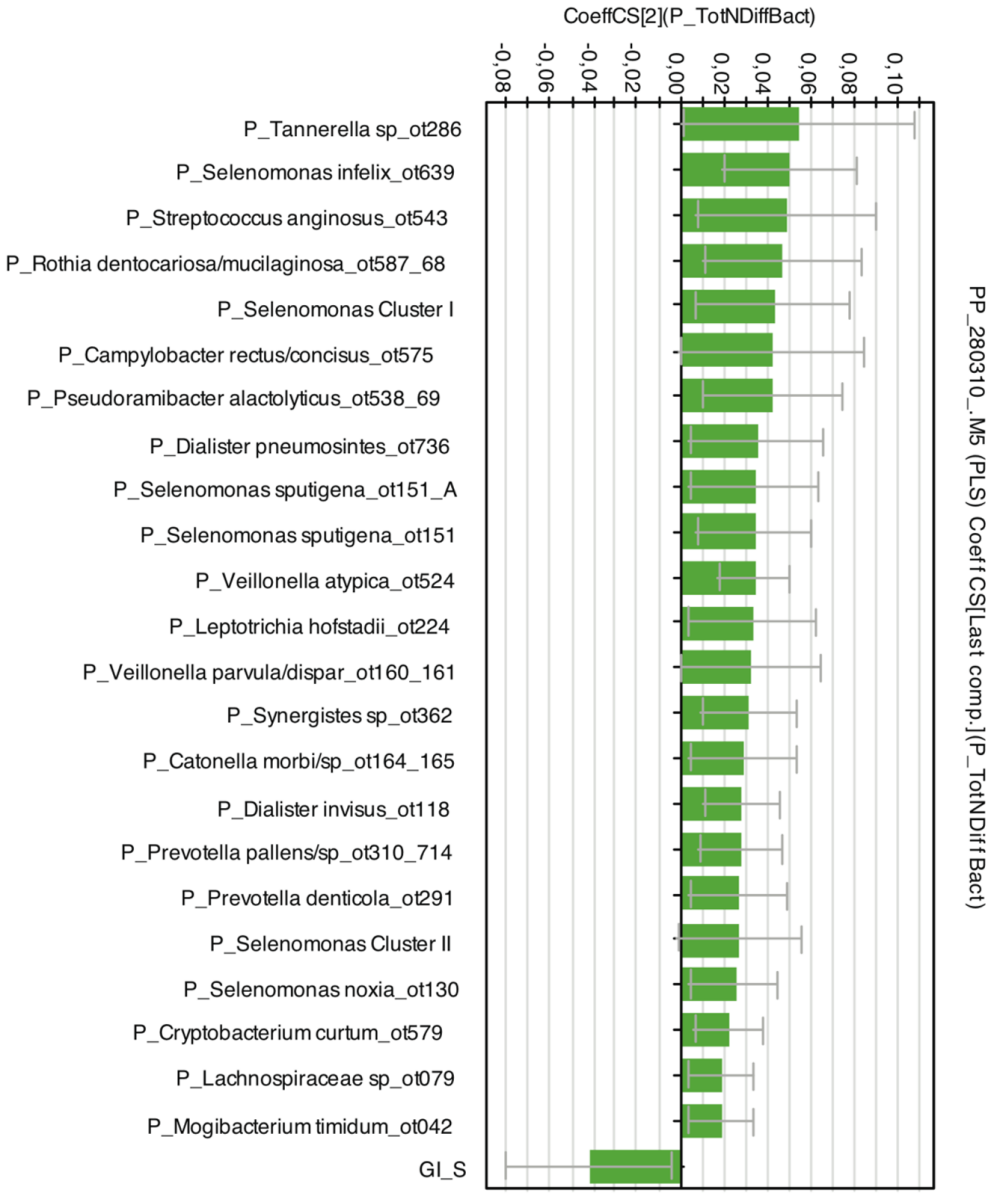
Contribution of bacterial and host-associated variables to bacterial species/phylotype diversity in subgingival PP samples. PLS regression coefficient plot identified X variables with statistically significant correlation to Y (Y  =  number of different bacterial species/phylotypes in PP samples). Among them those with coefficients >0.02 or <−0.02 are shown and bacterial species/phylotypes were subjected to phylogenetic analysis presented in Figure 4. The PLS model included 166 X variables. The model explained 98% and, according to cross validation, predicted 52% of the variation in Y. X variables were scaled and centered and Y variable scaled. The confidence intervals were derived from jack knifing. The coefficients are statistically significant when the error bars do not cross the 0 line. GCF: gingival crevicular fluid; PP: paper point.

To delineate differences between the bacterial species found in GCF and PP samples a phylogenetic tree (Figure 4) was constructed of the species/phylotypes that had statistically significant PLS regression coefficients (>0.02 or <−0.02) and thus predicted species/phylotype diversity in GCF samples (data from Figure 2C) and PP samples (data from Figure 5). From the 49 species/phylotypes included in the phylogenetic analysis, 38 (78%) were found in GCF samples, 23 (47%) in PP samples, and 12 (25%) in both. The species distribution among the 7 phyla or among all species/phylotypes did not significantly differ between GCF and PP samples in bivariate data analyses (Figure 4).

**Figure 6:**
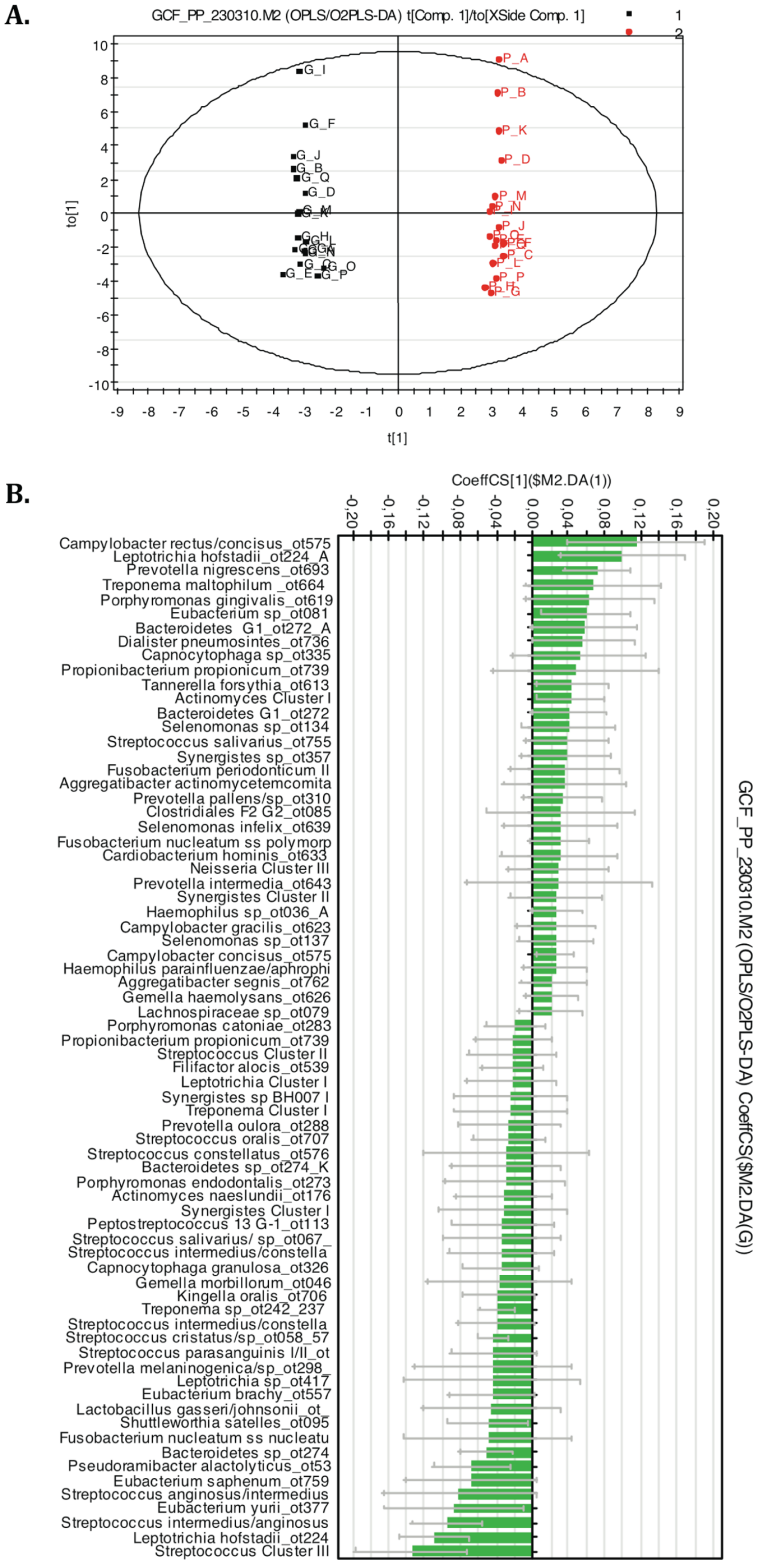
Bacterial species/phylotypes responsible for the difference in bacterial composition between GCF samples and PP samples. Multivariate OPLS/2OPLS-DA model was generated for 2 classes. The score plot (Panel A) demonstrates the relationship between the 2 types of samples and between the patients. Capital letters are patient identifiers, “G” (black color) for GCF samples and “P” (red color) for PP samples. The X variables included 133 species/phylotypes identified by DNA microarray. The binary Y variable defines the group membership to GCF or PP samples. Ellipse: Hotelling T2 (0.95). PLS regression analysis (Panel B) indicated the extent each variable positively or negatively contributed to GCF sample membership and *vice versa* to PP sample membership. The coefficients are statistically significant when the error bars do not cross the 0 line. The model explained 99% of the variation of Y and, according to cross validation, predicted 70% of the variation of Y. X variables were scaled and centered and Y variables scaled. The confidence intervals (95%) were derived from jack knifing. GCF: gingival crevicular fluid; PP: paper point.

A multivariate data analysis approach, quantitative OPLS/O2PLS-DA modeling, was applied to further examine differences in the bacterial species/phylotype composition between GCF and PP samples. In the model generated for 2 classes, all 133 bacterial species/phylotypes found in the GCF and PP samples were used as X variables (Figure 6). The model explained 99% of the variation related to the 2 sample groups. The score plot shows that the model clearly separated the GCF and PP samples (Figure 6A). Regression coefficients >0.02 or <−0.02 delineated the most important species/phylotypes (N = 34 and N = 38, respectively) responsible for the difference between GCF and PP sample groups (Figure 6B), the positive coefficient values signifying GCF samples and the negative values, PP samples. Among the species significantly contributing to the sample group separation, species/taxonomic groups traditionally classified to Gram-negative species were more frequent in GCF (10/12 [83%]) than PP samples (4/15 [27%]) (Figure 6B). The species/phylotypes with significant positive regression coefficients, thus representing the strongest contributors to GCF sample membership, included members of 5 phyla: *Bacteroidetes* (*Prevotella nigrescens, Tannerella forsythia, Bacteroidetes* G1 sp X083_ot272_X17, *Bacteroidetes* G1 sp X083_ot272_AA81), *Proteobacteria* (*Campylobacter rectus/concisus*, *C. concisus*, *Haemophilus* sp BJ095), *Fusobacteria* (*Fusobacterium nucleatum* ss *polymorphum, Leptotrichia hofstadii* FAC5_ot224_AA58), *Firmicutes* (*Dialister pneumosintes*, *Peptostreptococcaceae* [*11*] *[G-7]* BB142 sp*_*ot081) and *Actinobacteria* (*Actinomyces* Cluster I). Species/phylotypes or groups with significant negative regression coefficients, thus representing the strongest contributors to PP sample membership, also belonged to 5 phyla: *Firmicutes* (*Streptococcus* Cluster III, *S. intermedius/anginosus, S. anginosus/intermedius, S. intermedius/constellatus, S. parasanguinis, S. cristatus/*sp BM035 ot058, *Eubacterium yurii, Eubacterium saphenum, Eubacterium brachy, Pseudoramibacter alactolyticus* and *Shuttleworthia satelles*), *Proteobacteria* (*Kingella oralis), Fusobacteria* (*L. hofstadii* FAC5_ot224_Y55), *Bacteroidetes* (*Bacteroidetes* sp clone AU126_ot274) and *Spirochaetes* (*Treponema* sp AU076 ot242) (Figure 5B).

## Discussion

To our knowledge this is the first study to identify bacteria in GCF samples although host-derived constituents of GCF have been extensively studied during the past 40 years [9]. Interestingly, we found a wide diversity of different species in GCF samples despite the small sample amounts, from 4 to 12 ug per patient, and low bacterial concentrations, which could have challenged the yield of representative bacterial DNA for processing and DNA microarray analysis. The extracted DNA appeared to be of sufficient quality and quantity and the PCR amplification and microarray analysis were successful, as suggested by identification of 118 species belonging to 41 genera or higher taxa, altogether representing 8 phyla/candidate phyla in GCF samples. Bacterial species/phylotype diversity in GCF samples was comparable to that in subgingival PP samples obtained from the same periodontal sites as the GCF samples. Data on subgingival species diversity have been somewhat lower or of the same magnitude in other studies using 16S rDNA cloning [10], [13], [14], [19] and 16S rDNA microarray [14], [15].

In order to obtain representative bacterial samples for the present purposes, filter paper strips and paper points were chosen for sampling from GCF and periodontal pockets, respectively. Both devices absorb GCF with its various constituents e.g., bacteria, but the sampling procedure with paper points introduced along the root surface down to the bottom of the periodontal pockets additionally disrupts the attached subgingival biofilms, thus incorporating both unattached and attached subgingival bacteria in PP samples. Choosing these sampling methods instead of others, such as using capillary tubes for GCF and curettes for subgingival sampling, was based on the attempt to cause least possible stimulation and trauma to inflamed periodontal tissues and thus to minimize increase of GCF flow and gingival bleeding due to the mechanical sampling procedure. Bleeding by itself dilutes pocket contents and causes pressure in the pocket. In addition, strictly aseptic sampling technique and isolation of the sampling area were applied to avoid sample contamination from supragingival plaque and saliva.

The low patient number but large number of variables comprising demographic, systemic, periodontal and bacterial variables prompted us to use multivariate PLS modeling for the data analysis due to its capacity to handle complex data including low number of subjects, noise and artifacts as well as distribution and colinearity of variables [20]. Projection methods also enable effective data visualization tools that help interpret complex data and thereby also provide enhanced possibilities to comprehend implications. For the present data analyses PLS and OPLS/2OPLS-DA models with high predictive power were chosen according to the cross validation procedure described in the Methods section. Score and loading plots were used for an overview of the respective correlation matrices, and regression coefficients for prediction of responses, such as bacterial species/phylotype diversity and the GCF sample or PP sample membership. The coefficients for individual variables were generally low probably due to the complexity of the indigenous subgingival bacterial community. However, with use of confidence intervals to define statistical significance certain species were separated as the most important contributors to the responses in question. Regardless of the potent multivariate approach used for the analysis of the complex set of microbial and host variables, the questions concerning interindividual differences in GCF *versus* PP microbiota as well as time stability of microbial profiles still remain to be further investigated.

Our result that the statistically significant PLS regression coefficients distinguished a group of bacterial species/phylotypes among the diverse microbiota in GCF samples indicates that these species/phylotypes were the strongest contributors to and predictors for the species diversity in GCF samples. It also suggests that these species/phylotypes favored a multispecies environment. They appeared to be species traditionally classified as Gram-negative anaerobes belonging to genera *Campylobacter, Selenomonas*, *Porphyromonas*, *Catonella* and *Tannerella*, but also Gram-positive species from genera *Eubacterium, Peptostreptococcus* and *Streptococcus.* Thus, the species/phylotypes identified using PLS regression analysis as significant predictors for species/phylotype diversity in GCF samples mainly belonged to the late colonizers in dental plaque [21], and are known of their preferred growth in subgingival environment and their association with periodontitis [10], [11], [13], [15], [22]. Interestingly, milleri group streptococci, such as *S. intermedius, S. constellatus* and *S. anginosus,* related to abscesses and other pathologies, but also some mitis streptococci, known for their early plaque colonization and compatibility with dental health [23], [24], were detected among the species predicting bacterial species diversity in GCF samples. However, other early colonizers on tooth surfaces, such as *Actinomyces* spp., *Rothia dentocariosa, Gemella haemolysans, Eikenella corrodens*, *K. oralis* and *Haemophilus* spp. were not represented among the predictor species. *Fusobacterium* spp. that coaggregate with species colonizing early e.g., *Actinomyces* spp. and *Streptococcus* spp., but also with those colonizing late e.g., *P. gingivalis* and *Eubacterium* spp., in dental plaque [21], were not found among the positive predictors for species/phylotype diversity in GCF samples. This may mean that fusobacteria remained below the detection level or that they did not serve as a core for detached bacterial cell aggregates. Altogether, these results suggest that certain bacterial species mainly belonging to mature subgingival biofilms readily detach from subgingival biofilms or lyse/release DNA, both alternatives being able to cause host pathology in parenteral space.

None of the host-associated variables correlated significantly positively and only a single periodontal variable, number of teeth with CAL <4 mm, correlated significantly negatively to the species/phylotype diversity in GCF samples, suggesting that the healthier the periodontium the lower the species diversity. This is in accord with studies reporting lower number of different subgingival species in subjects with healthy periodontal conditions than in those with periodontitis [10], [15]. Somewhat unexpected was that host variables, particularly periodontal variables, did not more clearly correlate to bacterial diversity, although the robustness of the response variable could offer one explanation.

Although most of the 133 identified bacterial species were detected both in GCF and in PP samples some species, such as *E. saphenum, F. nucleatum* ss *nucleatum, S. sputigena* and *Treponema* Cluster I, frequent in traditional subgingival samples in periodontitis, were only found in GCF samples. These species could be prone to be flushed out in GCF or the result may have been affected by the generally low detection rate (*Treponema* Cluster I) in the present patients, dependence on the probes (*E. saphenum, S. sputigena*) or possibly that in PP samples they remained under detection level (*F. nucleatum* ss *nucleatum*). To exploit the potential of the full data set with quantitative bacterial data we subsequently applied OPLS/O2PLS discriminant analysis and found that certain species significantly positively or negatively contributed to GCF sample membership and vice versa to PP sample membership.

The positive contributors to GCF sample membership were mainly species traditionally classified as Gram-negative anaerobes, such as *C. rectus/concisus, P. nigrescens*, *D. pneumosintes* and *T. forsythia,* that are common subgingival microbiota and associated with periodontitis. Somewhat unexpected was that *Campylobacter* spp. were the only significantly contributing motile species. It could have been speculated that periodontitis-associated motile bacteria actively leave biofilm and will thus be associated with GCF sample. One possible reason is that other motile bacteria remained under detection level of HOMIM analysis that discloses predominant bacteria in the sample. As seen in the results, several motile species among *Selenomonas* and *Treponema* genera were positively associated with GCF sample, but the association did not reach statistical significance.

On the other hand, the positive contributors to PP sample membership included mainly Gram-positive bacteria, such as mitis- and milleri-group streptococci and anaerobic rods, all of them current or previous members of genus *Eubacterium* (*E. brachy, E. saphenum*, *E. yurii*, *S. satelles* and *P. alactolyticus*). As mitis streptococci are early plaque colonizers and common in periodontal health [25], [26] less is known of *S. satelles* [27] that recently was associated with periodontal destruction [15]. *P. alactolyticus,* former *Eubacterium alactolyticum* [28], has been found in subgingival samples of periodontally healthy children [29] but also in those of periodontitis patients [15]. The species separation between GCF and PP samples supports the capability of the employed sampling methods to collect unattached and attached microbiota. For example, Gram-negative species may occur unattached or loosely attached in periodontal pockets and were more readily absorbed with GCF to filter paper strips than the above-mentioned Gram-positive species.

Our results suggest that in periodontal pockets there are unattached bacteria and/or their species-specific DNA that can be identified from GCF samples taken at the orifice of the gingival sulcus and thus without entering the periodontal pockets. To date data are accumulating of passive and active mechanisms for bacterial dispersal from biofilms [16], [30], but little knowledge is still available of the life cycle of complex natural biofilms such as human subgingival dental plaque. This is an interesting research field since the quality and quantity of bacterial species detached from subgingival biofilms may differ in different phases of periodontal inflammation. Thus, in future studies, monitoring the bacterial composition of GCF may give information of the current activity of the subgingival biofilm *in vivo*. Based on the present results, we conclude that the DNA microarray analysis of GCF samples from periodontitis patients revealed phylogenetically diverse bacterial populations and that species traditionally classified as Gram-negative anaerobes appeared to be the main predictors for the species diversity as well as for the distinction of GCF from PP samples.

## Materials and Methods

### Ethics statement

The study design was approved by the Ethics Committee of the Faculty of Medicine for Human Studies, Gazi University (June 16, 2006, #208) and the patients signed an informed consent.

### Patients

Seventeen adult patients with periodontitis (age range 33–64 years), who met the inclusion criteria and volunteered for the study, were recruited from the Department of Periodontology, Gazi University, Ankara, Turkey (June-August 2008).

Data on demographics and smoking habit were collected with a self-reported questionnaire. Body Mass Index (BMI) was calculated. Blood samples for leukocyte count and fasting blood glucose were taken and analyzed at the Gazi University Hospital Laboratory.

The diagnosis of periodontitis was based on clinical and radiographic examination. The study inclusion criteria were as follows: generally healthy adults, at least 5 teeth with clinical attachment level (CAL) exceeding 4 mm, no continuous medications, no mechanical periodontal therapy or antibiotics during the preceding 6 months, and never had periodontal surgery. Plaque Index (PlI), Gingival Index (GI), probing depths (PD), bleeding on probing (BOP), and clinical attachment level (CAL) were recorded at 6 sites (both vestibular and oral aspects of the mesial, distal and mid surfaces) per tooth from all teeth in the dentition by a periodontist, as previously described [31].

### Bacterial sampling

Six caries-free periodontal sites with the deepest, inflamed periodontal pockets and attachment loss were selected for bacterial sampling during the clinical examination of the patients. One week later, the GCF samples and subgingival PP samples were taken as follows: After removing supragingival plaque with sterile curettes using coronal strokes starting from the gingival margin, a PerioPaper strip (Oraflow Inc.) was gently placed to the orifice of the gingival sulcus to collect fluid approximately half of the strip length. Care was taken not to contaminate the strip from the supragingival tooth surfaces or saliva, or to mechanically irritate the gingiva. Approximately 10 min after the GCF sampling, subgingival samples were taken with sterile paper points (MetaAbsorbent Paperpoints; MetaBiomed Co. Ltd., Chungbuk, Korea) from the same 6 periodontal sites as the GCF samples. The PP samples were introduced to the bottom of each periodontal pocket and removed after 10 sec [32]. The GCF samples and the PP samples were transferred to separate sterile Eppendorf tubes. For each patient, one GCF sample pooled from 6 sites and one PP sample pooled from 6 pockets were immediately placed at −70°C, where they were preserved until sent to the Department of Oral Microbiology, Umeå University, Sweden, for DNA extraction. To quantify the samples, eppendorf tubes with strips and those with paper points were weighed before and after sampling.

### DNA extraction and 16S rRNA gene amplification for DNA microarray analysis

For DNA extraction, bacteria were eluted from 6 PerioPaper strips (GCF) and from 6 paper points (subgingival sample), respectively, per patient using 400 ml buffer (1×PBS or TE). Genomic DNA was then extracted using the QiaAmp DNA isolation kit (QiaGen), according to the instructions of the manufacturer. 16S rRNA genes were amplified from each genomic DNA sample, isolated as above, by means of a double PCR approach, essentially as described earlier [15]. For this, 2 separate 25 µl PCR reactions were run, using an equimolar mixture of oligonucleotides 4F:59 and 6F:59 (each at a final concentration of 200 nM) as the forward primer, and oligonucleotides 1541R and 1492R, respectively (final concentration 400 nM) as reverse primers. The reaction mixtures also contained 1× PCR buffer II and 1.5 U AmpliTaq® DNA polymerase (Applied Biosystems), 1.5 mM MgCl_2_, 200 nM of each deoxynucleotide, and 2 µl of the DNA template. The PCR cycling conditions were as previously described [15], with the exception that 72°C was used for the elongation steps instead of 68°C. The 2 PCR reactions were pooled, purified using the QiaQuick PCR purification kit (QiaGen), and dried down using Speedvac, prior to microarray analysis (HOMIM, Boston, MA) as previously described [15].

Oral taxon (OT) designation for each species/phylotype are provided in Human Oral Microbiome Database (HOMD) www.homd.org. The HOMD links sequence data of each OT with phenotypic, phylogenetic, clinical and bibliographic information. The HOMD also provides information of the OTs included in each species cluster.

### Statistical data analysis

For the present data analysis, traditional statistics did not seem to be optimal due to the low number of patients and colinearity of the variables. Therefore, a multivariate regression technique, PLS (partial least squares) projections to latent structures [20], was used for modeling, analysis and interpretation of the bacterial and host-associated data. OPLS/O2PLS-DA **(**orthogonal PLS discriminant analysis) [33], a new supervised classification analysis tool, was used to quantitatively analyze the contribution of each species/phylotype (X variables) to the class separation by creating 2 dummy Y variables, one for GCF samples and another for subgingival PP samples.

The study variables were imported into the SIMCA-P+12 software package (Umetrics Inc.) for the data analysis and visualization. The quantitative bacterial data were scored from 1 to 5. Data were imported as continuous variables for host-associated variables (age, BMI, leukocyte count and glucose levels) and periodontal variables (means per subject of PlI, GI, BOP, PD and CAL, number of teeth, number of different bacterial species or groups per sample and weight of the GCF and PP samples) and as categorical variables (number of teeth with 3 severity categories for PD and CAL, gender, socioeconomic status [low, intermediate, high] and smoking habit [never, stopped, current]). After centering and scaling to unit variance the data were analyzed. To measure the predictive power of the model, cross validation procedure contained the following: parts of the data were kept out of model development, the kept-out parts were predicted by the model and predictions of the kept-out parts were compared with the obtained actual values. Several tools were used to visualize the results. Score plots provided correlation patterns among the patients and among the samples. Loading scatter plots revealed the impact of each X variable on the formation of the scores, since the loading scatter plots are complementary and superimposable to the respective score plots. PLS regression coefficients for the variables showed the extent each X variable contributed to Y. The precision was derived from confidence interval (95%) using jack knifing. Statistical significance prevails if the error bars in the figures do not cross the 0 line.

### Data accession

The data discussed in this publication have been deposited in a MIAME compliant database, NCBI's Gene Expression Omnibus [34] and are accessible through GEO Series accession number GSE21929 (http://www.ncbi.nlm.nih.gov/geo/query/acc.cgi?acc=GSE21929).

## Supporting Information

Table S1 The 133 bacterial species/phylotypes found in crevicular fluid and/or subgingival paper point samples and their respective number codes assigned to be used in Figure 2A.
(0.13 MB DOC)

## References

[1] Baelum V, van Palenstein Helderman W, Hugoson A, Yee R, Fejerskov O (2007). A global perspective on changes in the burden of caries and periodontitis: implications for dentistry.. J Oral Rehabil.

[2] Loos BG (2005). Systemic markers of inflammation in periodontitis.. J Periodontol.

[3] Libby P (2002). Inflammation in atherosclerosis.. Nature.

[4] Ridker PM, Silvertown JD (2008). Inflammation, C-reactive protein, and atherothrombosis.. J Periodontol.

[5] Karched M, Ihalin R, Eneslatt K, Zhong D, Oscarsson J (2008). Vesicle-independent extracellular release of a proinflammatory outer membrane lipoprotein in free-soluble form.. BMC Microbiol.

[6] Oscarsson J, Karched M, Thay B, Chen C, Asikainen S (2008). Proinflammatory effect in whole blood by free soluble bacterial components released from planktonic and biofilm cells.. BMC Microbiol.

[7] Lamster IB, Ahlo JK (2007). Analysis of gingival crevicular fluid as applied to the diagnosis of oral and systemic diseases.. Ann N Y Acad Sci.

[8] Nakashima K, Giannopoulou C, Andersen E, Roehrich N, Brochut P (1996). A longitudinal study of various crevicular fluid components as markers of periodontal disease activity.. J Clin Periodontol.

[9] Armitage GC (2004). Analysis of gingival crevice fluid and risk of progression of periodontitis.. Periodontol 2000.

[10] Paster BJ, Boches SK, Galvin JL, Ericson RE, Lau CN (2001). Bacterial diversity in human subgingival plaque.. J Bacteriol.

[11] Kumar PS, Griffen AL, Moeschberger ML, Leys EJ (2005). Identification of candidate periodontal pathogens and beneficial species by quantitative 16S clonal analysis.. J Clin Microbiol.

[12] de Lillo A, Ashley FP, Palmer RM, Munson MA, Kyriacou L (2006). Novel subgingival bacterial phylotypes detected using multiple universal polymerase chain reaction primer sets.. Oral Microbiol Immunol.

[13] Faveri M, Mayer MP, Feres M, de Figueiredo LC, Dewhirst FE (2008). Microbiological diversity of generalized aggressive periodontitis by 16S rRNA clonal analysis.. Oral Microbiol Immunol.

[14] Preza D, Olsen I, Willumsen T, Boches SK, Cotton SL (2009). Microarray analysis of the microflora of root caries in elderly.. Eur J Clin Microbiol Infect Dis.

[15] Colombo AP, Boches SK, Cotton SL, Goodson JM, Kent R (2009). Comparisons of subgingival microbial profiles of refractory periodontitis, severe periodontitis, and periodontal health using the human oral microbe identification microarray.. J Periodontol.

[16] Kaplan JB Biofilm dispersal: mechanisms, clinical implications, and potential therapeutic uses.. J Dent Res.

[17] Costerton JW, Stewart PS, Greenberg EP (1999). Bacterial biofilms: a common cause of persistent infections.. Science.

[18] Furukawa S, Kuchma SL, O'Toole GA (2006). Keeping their options open: acute versus persistent infections.. J Bacteriol.

[19] Aas JA, Paster BJ, Stokes LN, Olsen I, Dewhirst FE (2005). Defining the normal bacterial flora of the oral cavity.. J Clin Microbiol.

[20] Eriksson L, Antti H, Gottfries J, Holmes E, Johansson E (2004). Using chemometrics for navigating in the large data sets of genomics, proteomics, and metabonomics (gpm).. Anal Bioanal Chem.

[21] Kolenbrander PE, Palmer RJ, Rickard AH, Jakubovics NS, Chalmers NI (2006). Bacterial interactions and successions during plaque development.. Periodontol 2000.

[22] Socransky SS, Haffajee AD (1992). The bacterial etiology of destructive periodontal disease: current concepts.. J Periodontol.

[23] Dige I, Nyengaard JR, Kilian M, Nyvad B (2009). Application of stereological principles for quantification of bacteria in intact dental biofilms.. Oral Microbiol Immunol.

[24] Socransky SS, Manganiello AD, Propas D, Oram V, van Houte J (1977). Bacteriological studies of developing supragingival dental plaque.. J Periodontal Res.

[25] Slots J (1977). Microflora in the healthy gingival sulcus in man.. Scand J Dent Res.

[26] Marsh PD (1994). Microbial ecology of dental plaque and its significance in health and disease.. Adv Dent Res.

[27] Downes J, Munson MA, Radford DR, Spratt DA, Wade WG (2002). *Shuttleworthia satelles* gen. nov., sp. nov., isolated from the human oral cavity.. Int J Syst Evol Microbiol.

[28] Willems A, Collins MD (1996). Phylogenetic relationships of the genera *Acetobacterium* and *Eubacterium* sensu stricto and reclassification of *Eubacterium alactolyticum* as *Pseudoramibacter alactolyticus* gen. nov., comb. nov.. Int J Syst Bacteriol.

[29] Kamma JJ, Diamanti-Kipioti A, Nakou M, Mitsis FJ (2000). Profile of subgingival microbiota in children with mixed dentition.. Oral Microbiol Immunol.

[30] Karatan E, Watnick P (2009). Signals, regulatory networks, and materials that build and break bacterial biofilms.. Microbiol Mol Biol Rev.

[31] Dogan B, Buduneli E, Emingil G, Atilla G, Akilli A (2005). Characteristics of periodontal microflora in acute myocardial infarction.. J Periodontol.

[32] Dogan B, Antinheimo J, Cetiner D, Bodur A, Emingil G (2003). Subgingival microflora in Turkish patients with periodontitis.. J Periodontol.

[33] Wiklund S, Johansson E, Sjostrom L, Mellerowicz EJ, Edlund U (2008). Visualization of GC/TOF-MS-based metabolomics data for identification of biochemically interesting compounds using OPLS class models.. Anal Chem.

[34] Edgar R, Domrachev M, Lash AE (2002). Gene Expression Omnibus: NCBI gene expression and hybridization array data repository.. Nucleic Acids Res.

