# Supplementary material for: Specified Species in Gingival Crevicular Fluid Predict Bacterial Diversity
Source: PLoS One. 2010 Oct 25;5(10):e13589. doi: 10.1371/journal.pone.0013589 (PMC2963608; doi:10.1371/journal.pone.0013589)
Supplement: Table S1 — The 133 bacterial species/phylotypes found in crevicular fluid and/or subgingival paper point samples and their respective number codes assigned to be used in Figure 2A. (0.13 MB DOC) [file pone.0013589.s001.doc]

| Bacterial species/phylotype | Assigned number | |
| --- | --- | --- |
| *Actinomyces* Cluster I | | 1 |
| *Actinomyces gerencseriae*_ot618 | | 2 |
| *Actinomyces naeslundii*_ot176 | | 3 |
| *Actinomyces viscosus*_ot688 | | 4 |
| *Aggregatibacter actinomycetemcomitans*_ot531 | | 5 |
| *Aggregatibacter segnis*_ot762 | | 6 |
| *Atopobium rimae*_ot754_K24 | | 7 |
| *Atopobium rimae*_ot754_X12 | | 8 |
| *Bacteroidetes* sp_ot274_K76 | | 9 |
| *Bacteroidetes* sp_ot274_X17 | | 10 |
| *Bacteroidetes* G1 sp_ot272_AA81 | | 11 |
| *Bacteroidetes* G1 sp_ot272_X17 | | 12 |
| *Campylobacter* Cluster I | | 13 |
| *Campylobacter concisus*_ot575 | | 14 |
| *Campylobacter gracilis*_ot623 | | 15 |
| *Campylobacter rectus*/*concisus*_ot575_748 | | 16 |
| *Campylobacter showae*_ot763 | | 17 |
| *Capnocytophaga granulosa*_ot326 | | 18 |
| *Capnocytophaga* sp_ot332 | | 19 |
| *Capnocytophaga* sp X066_ot335 | | 20 |
| *Capnocytophaga sputigena*_ot775 | | 21 |
| *Cardiobacterium hominis*_ot633 | | 22 |
| *Cardiobacterium valvulum*_ot540 | | 23 |
| *Catonella morbi*/sp_ot164_165 | | 24 |
| *Clostridiales* F2 G2 sp_ot085 | | 25 |
| *Cryptobacterium curtum*_ot579 | | 26 |
| *Desulfobulbus* sp_ot041 | | 27 |
| *Dialister invisus*_ot118 | | 28 |
| *Dialister pneumosintes*_ot736 | | 29 |
| *Eubacterium brachy*_ot557 | | 30 |
| *Eubacterium nodatum*_ot694 | | 31 |
| *Eubacterium saburreum*_ot494 | | 32 |
| *Eubacterium saphenum* I | | 33 |
| *Eubacterium saphenum*_ot759 | | 34 |
| *Eubacterium* sp_ot081 | | 35 |
| *Eubacterium yurii*_ot377 | | 36 |
| *Filifactor alocis*_ot539 | | 37 |
| *Fusobacterium nucleatum* ss *polymorphum* I | | 38 |
| *Fusobacterium nucleatum* ss *nucleatum*_ot598 | | 39 |
| *Fusobacterium periodonticum* II | | 40 |
| *Gemella haemolysans*_ot626 | | 41 |
| *Gemella morbillorum*_ot046 | | 42 |
| *Granulicatella adiacens*/*elegans*_ot534_596 | | 43 |
| *Haemophilus* sp_ot036_AA97 | | 44 |
| *Haemophilus* sp_ot036_K64 | | 45 |
| *Haemophilus parainfluenzae*/*aphrophilus* I | | 46 |
| *Kingella oralis*_ot706 | | 47 |
| *Lachnospiraceae* sp_ot079 | | 48 |
| *Lactobacillus gasseri*/*johnsonii*_ot_615_819 | | 49 |
| *Lautropia mirabilis*_ot022 | | 50 |
| *Leptotrichia* Cluster I | | 51 |
| *Leptotrichia hofstadii*_ot224_AA58 | | 52 |
| *Leptotrichia hofstadii*_ot224_Y55 | | 53 |
| *Leptotrichia* sp_ot417 | | 54 |
| *Megasphaera micronuciformis*_ot122 | | 55 |
| *Megasphaera* sp_ot123 | | 56 |
| *Mogibacterium timidum*_ot042 | | 57 |
| *Neisseria* Cluster III | | 58 |
| *Neisseria flavescens* I | | 59 |
| *Neisseria* Cluster IV | | 60 |
| *Parvimonas micra* I_ot111 | | 61 |
| *Peptostreptococcus* 13 G-1 sp_ot113 | | 62 |
| *Peptostreptococcus stomatis*_ot112 | | 63 |
|  | |  |
|  | |  |
|  | |  |
|  | |  |
|  | |  |
|  | |  |
| *Porphyromonas catoniae*_ot283 | | 64 |
| *Porphyromonas endodontalis*_ot273 | | 65 |
| *Porphyromonas gingivalis*_ot619 | | 66 |
| *Prevotella* Cluster I | | 67 |
| *Prevotella denticola*_ot291 | | 68 |
| *Prevotella intermedia*_ot643 | | 69 |
| *Propionibacterium propionicum*_ot739 I_AB71 | | 76 |
| *Propionibacterium propionicum*_ot739 I_AB72 | | 77 |
| *Pseudoramibacter alactolyticus*_ot538_AB69 | | 78 |
| *Pseudoramibacter alactolyticus*_ot538_AB70 | | 79 |
| *Rothia dentocariosa*/*mucilaginosa*_ot587_681 | | 80 |
| *Selenomonas* Cluster I | | 81 |
| *Selenomonas infelix*_ot639 | | 82 |
| *Selenomonas noxia*_ot130 | | 83 |
| *Selenomonas* sp_ot134 | | 84 |
| *Selenomonas* sp_ot137 | | 85 |
| *Selenomonas* sp/DS071_ot138_146 | | 86 |
| *Selenomonas sputigena*_ot151_AB04 | | 87 |
| *Selenomonas sputigena*_ot151_K65 | | 88 |
| *Selenomonas artemidis*_ot124 | | 89 |
| *Selenomonas Cluster* II | | 90 |
| *Shuttleworthia satelles*_ot095 | | 91 |
| *Solobacterium moorei*_ot678 | | 92 |
| *Streptococcus anginosus*/*gordonii*_ot543_622 | | 93 |
| *Streptococcus anginosus*/*intermedius*_ot543_644 | | 94 |
| *Streptococcus anginosus*_ot543 | | 95 |
| *Streptococcus* Cluster II | | 96 |
| *Streptococcus* Cluster III | | 97 |
| *Streptococcus constellatus*_ot576 | | 98 |
| *Streptococcus cristatus/*sp_ot058_578 | | 99 |
| *Streptococcus gordonii*/*anginosus*_ot543_622 | | 100 |
| *Streptococcus infantis*/*cristatus*_ot578_638 | | 101 |
| *Streptococcus intermedius*/*anginosus*_ot543_644 | | 102 |
| *Streptococcus intermedius*/*constellatus*_ot576_644 | | 103 |
| *Streptococcus intermedius*/*constellatus*_ot576_644 | | 104 |
| *Streptococcus mitis* bv2/sp_ot069_398 | | 105 |
| *Streptococcus oralis*_ot707 | | 106 |
| *Streptococcus parasanguinis*_ot411_721 | | 107 |
| *Streptococcus salivarius*/sp_ot067_755 | | 108 |
| *Streptococcus salivarius*_ot755 | | 109 |
| *Streptococcu*s *sanguinis*_ot758_AB75 | | 110 |
| *Streptococcus sanguinis*_ot758_AB78 | | 111 |
| *Streptococcus* sp/sp_ot070_071 | | 112 |
| *Streptococcus* Cluster I | | 113 |
| *Streptococcus australis*_ot073 | | 114 |
| *Streptococcu*s *mutans*_ot686 | | 115 |
| *Synergistes* Cluster I | | 116 |
| *Synergistes* Cluster II | | 117 |
| *Synergistes* sp_ot362 | | 118 |
| *Synergistes* sp_ot357 | | 119 |
| *Synergistes* sp_ot358 | | 120 |
| *Synergistes* sp BH007 I | | 121 |
| *Synergistes* sp W090 II | | 122 |
| *Tannerella forsythia*_ot613 | | 123 |
| *Tannerella* sp_ot286 | | 124 |
| TM7 G-1 sp/sp_ot349_346 | | 125 |
| TM7 sp_ot356 | | 126 |
| *Treponema* Cluster I | | 127 |
| *Treponema maltophilum*_ot664 | | 128 |
| *Treponema* sp/sp_ot242_237 | | 129 |
| *Veillonella atypica*_ot524 | | 130 |
| *Veillonella* Cluster II | | 131 |
| *Veillonella parvula*/*dispar*_ot160_161 | | 132 |
| *Veillonella parvula*_ot161 | | 133 |
